# The Quality of Clinical Maternal and Neonatal Healthcare – A Strategy for Identifying ‘Routine Care Signal Functions’

**DOI:** 10.1371/journal.pone.0123968

**Published:** 2015-04-15

**Authors:** Stephan Brenner, Manuela De Allegri, Sabine Gabrysch, Jobiba Chinkhumba, Malabika Sarker, Adamson S. Muula

**Affiliations:** 1 Institute of Public Health, Ruprecht-Karls-University, Heidelberg, Germany; 2 Department of Community Health, University of Malawi, College of Medicine, Blantyre, Malawi; Taipei Medical University, TAIWAN

## Abstract

**Background:**

A variety of clinical process indicators exists to measure the quality of care provided by maternal and neonatal health (MNH) programs. To allow comparison across MNH programs in low- and middle-income countries (LMICs), a core set of essential process indicators is needed. Although such a core set is available for emergency obstetric care (EmOC), the ‘EmOC signal functions’, a similar approach is currently missing for MNH routine care evaluation. We describe a strategy for identifying core process indicators for routine care and illustrate their usefulness in a field example.

**Methods:**

We first developed an indicator selection strategy by combining epidemiological and programmatic aspects relevant to MNH in LMICs. We then identified routine care process indicators meeting our selection criteria by reviewing existing quality of care assessment protocols. We grouped these indicators into three categories based on their main function in addressing risk factors of maternal or neonatal complications. We then tested this indicator set in a study assessing MNH quality of clinical care in 33 health facilities in Malawi.

**Results:**

Our strategy identified 51 routine care processes: 23 related to initial patient risk assessment, 17 to risk monitoring, 11 to risk prevention. During the clinical performance assessment a total of 82 cases were observed. Birth attendants’ adherence to clinical standards was lowest in relation to risk monitoring processes. In relation to major complications, routine care processes addressing fetal and newborn distress were performed relatively consistently, but there were major gaps in the performance of routine care processes addressing bleeding, infection, and pre-eclampsia risks.

**Conclusion:**

The identified set of process indicators could identify major gaps in the quality of obstetric and neonatal care provided during the intra- and immediate postpartum period. We hope our suggested indicators for essential routine care processes will contribute to streamlining MNH program evaluations in LMICs.

## Introduction

Maternal and neonatal deaths are largely preventable, but still very common in low- and middle-income countries (LMICs). In 2013, the maternal mortality ratio (MMR) for developed nations was estimated at 16 deaths per 100,000 live births, but as high as 230 deaths per 100,000 live births for developing regions, and highest in Sub-Saharan Africa (SSA) with 510 maternal deaths per 100,000 live births [1]. Similar patterns are found for early (0–6 days of life) and late (7–28 days of life) neonatal mortality rates (NMR). In 2013 NMR estimates ranged from 2 deaths per 1,000 live births for early and less than 1 death per 1,000 live births for late neonatal mortality in developed nations, while these rates for developing regions were estimated as 13 and 4 deaths per 1,000 live births, respectively; with 20 and almost 7 deaths per 1,000 live births, respectively in the SSA region [2].

The majority of maternal deaths in LMICs are due to direct causes, such as postpartum hemorrhage (PPH), pregnancy-induced hypertension (i.e. new onset arterial hypertension in a pregnant woman after 20 weeks of gestation), or septic infections [3]. More than 40% of maternal deaths due to direct causes occur during the intrapartum period; of all maternal deaths during the postpartum period, 45% occur within the first 24 hours after delivery (i.e. early postpartum period) [4][5]. The majority of early neonatal deaths in LMICs is due to emergencies resulting from birth-related complications, such as birth asphyxia (i.e. ta newborn’s failure to initiate or maintain regular breathing at birth due to various causes), prematurity, and septic infections [6]. Between 25–45% of neonatal deaths occur within the first 24 hours, and up to 90% within the first 48 hours of newborn life [7][8].

**Fig 1:**
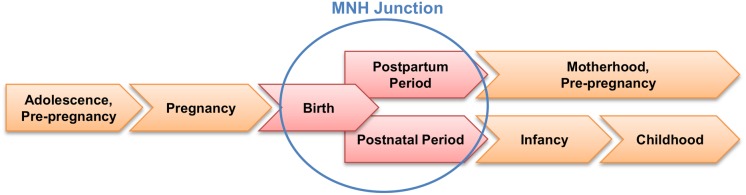
Continuum of Care and Maternal-Neonatal Health (MNH) Junction. Based on the Partnership for Maternal, Newborn & Child Health [63].

In response to this high burden of maternal and neonatal deaths, global strategies were developed to enable better integration of MNH services into the reproductive health agenda of LMICs [9]. The *continuum of care* framework represents such a strategic approach by interlinking healthcare services for women and newborns throughout pregnancy, childbirth, and infancy (see Fig 1) [10]. Along this continuum of care, one of the most critical moments is the junction between childbirth, postpartum and newborn care, which for the purpose of this article we labelled the *maternal-newborn health junction* (MNH Junction). This junction comprises the labor, delivery, immediate postpartum and immediate postnatal periods, which together represent the longest uninterrupted time interval of direct provider-patient interaction within the continuum of care. Assuming a pregnant woman arrives at a maternity unit around the onset of labor and remains in facility-based care until 24 to 48 hours after delivery, this interval spans over 2–4 days. Compared to other reproductive health services provided along the continuum of care, the MNH Junction offers the unique opportunity for non-fragmented and comprehensive MNH care during one of the most critical moments in the life of pregnant women and their newborns in terms of their mortality risks [11][12].

Clinically, patient management during the intrapartum, immediate postpartum and postnatal periods can be divided into routine and emergency care processes [13]. *Emergency care* refers to clinical processes that need to be provided rapidly in order to medically manage or stabilize a patient with a life-threatening complication. Emergency care processes therefore describe clinical responses to a health situation where a patient’s life is in imminent danger. Besides a number of stabilizing and life-saving clinical interventions, obstetric and neonatal emergency care also includes the timely arrangement for effective referral of mother or newborn to higher levels of care [14][15]. *Routine care* in contrast refers to clinical processes indicated in the care of every non-acute patient, and includes interventions such as essential treatments or clinical follow-ups. Routine care processes have their main purpose in evaluating and controlling a patient’s individual health risk, and thus allow the early detection and prevention of emergency situations. In obstetric and neonatal care, routine care refers to the identification, monitoring, and management of non-complicated pregnancies and deliveries [14].

In most LMICs, especially in remote rural areas where the availability of highly qualified staff is limited, both routine and emergency obstetric care functions are performed by the same cadre of clinical mid-level providers (i.e. midwives and nurse-midwives). T Their clinical competence in determining a patient’s health risk and managing life-threatening situations is therefore crucial for reducing maternal and neonatal mortality [16][17]. But the success of emergency care interventions, such as fluid replacement for hemorrhage, also depends on functional referral systems ensuring timely access to definitive higher-level care, such as obstetric surgery or blood transfusion [18][19]. In settings with weak referral structures or limited surgical coverage, such as in most rural LMICs, maternal and newborn mortality is often higher at the hospital level due to late referrals from lower level health centers[18]. In such settings, birth attendants’ clinical competence in both risk assessment and risk prevention as part of a systematically applied routine care performance is crucial to the identification and stabilization of complicated cases and in ensuring that definitive care arrangements are mead in a timely manner[9].

Over the past years and in relation to achieving Millennium Development Goals 4 and 5, strong international attention has been given to the definition, implementation, and evaluation of indicators for obstetric and neonatal emergency care processes [20][21]. Despite evidence on the impact of different obstetric and neonatal emergency interventions in LMICs [22][23], only a sub-set of key emergency interventions—the so-called Emergency Obstetric Care (EmOC) signal functions—have been identified as essential process of care indicators [21]. Primarily developed to assess the need for EmOC, these indicators are now consistently used in assessing MNH programs across resource-limited settings. With some suggestions existing already [24], a similarly well-defined key set of process indicators for obstetric and newborn routine care is yet to be determined,. Compared to EmOC, the number of available obstetric and newborn routine care process indicators appears endless considering the different dimensions of MNH [25][26]. While some indicators cover common aspect of routine care in general (e.g. infection prevention, interpersonal communication, prevention of eclampsia, partograph use, active management of third stage of labor (AMTSL), immediate newborn care, etc.), others are primarily focused on routine care processes only relevant to some obstetric and newborn patients (e.g. prevention of mother-to-child transmission of HIV, care for low-birth-weight infants) [27–32].

To comprehensively incorporate this variety of indicators is a challenge to MNH program evaluations. For instance, in their multi-country assessment of the quality of care for prevention and management of common maternal and newborn complications, the Maternal and Child Health Integrated Program (MCHIP) groups routine care process indicators relevant to labor and delivery into eight categories (‘initial client assessment’, ‘obstructed labor prevention’, ‘infection prevention’, ‘respectful care’, ‘immediate and essential newborn care’, ‘non-indicated or non-beneficial practices’, ‘AMTSL’, ‘pre-eclampsia screening’) [33–39]. Using methods of direct observation, each category consists of about 5–10 individual process indicators. Such comprehensive assessments are however not always feasible to smaller MNH programs in need of periodic monitoring and evaluation (M&E) [40][41]. Further work is required to identify which core process measures are most relevant to ongoing assessments of routine care practices in MNH programs.

A proposal of “new signal functions” published in 2012 [24], based on existing evidence and an expert survey, included a set of six routine care signal functions, three obstetric (partograph, infection prevention, AMTSL) and three related to the newborn (thermal protection, immediate breastfeeding, infection prevention including hygienic cord care). Some of these functions consist of several components (e.g. AMTSL) and evaluating their performance will thus require several questions or observation items, depending on the depth of the assessment. A study in Ghana used these new functions to assess the quality of routine and emergency obstetric and newborn care in all 64 delivery facilities in 7 districts of the Brong Ahafo Region [42]. The scale of that study and the low number of deliveries in most facilities did not permit observation, and the authors thus used provider-reported performance of the functions, validated by tracer items as well as health professional numbers and reported skills.

In this article, we attempt to work towards identifying a key set of MNH routine care process indicators that can be used for in-depth quality assessments relying on direct observation methods. We suggest and test a rationale for the selection of routine care process of care indicators essential to the MNH Junction that is rooted in both the epidemiology of maternal and neonatal mortality causes, as well as in the evidence-base of clinical obstetric care. Using data from a recent routine care process assessment conducted in Malawi, we then evaluate how well our indicator set performed in terms of applicability and information gain in a LMICS setting.

## Methods

### Study setting

Malawi is a country in SSA with a relatively high MMR and NMR (510/100,000 live births and 23/1,000 live births in 2013, respectively) [43]. The Ministry of Health currently implements a results-based financing program on MNH quality in Malawi (RBF4MNH Initiative). To determine the effect of this program, an impact evaluation was designed that also included a component to assess the clinical quality of routine MNH care [44]. To this end, we developed a process indicator selection rationale which will be further described below. In this article, we use findings from the impact evaluation’s baseline survey in April 2013, with the exclusive aim of illustrating the practicability of our conceptual process indicator selection approach to measuring processes of care at the MNH junction. This cross-sectional process of care assessment included 33 rural health facilities offering labor and delivery care. These 33 facilities are evenly distributed over four districts in the central (Dedza, Mchinji, Ntcheu) and southern regions (Balaka) of the country. We only included facilities providing EmOC (5 hospitals providing comprehensive EmOC (CEmOC) and 28 health centers providing basic EmOC (BEmOC)). All 33 facilities are also included in the overall impact evaluation design [44].

### Data collection and analysis

The data on quality of routine care processes was collected through non-participatory clinical observation of delivery cases during the intrapartum- and immediate postpartum period. We only included women presenting without complications to ensure comparability between cases in terms of clinical acuteness. Data were collected by local research assistants with professional backgrounds in midwifery using structured clinical observation checklists. These paper-based checklists collected information on 51 clinical routine care processes identified by our selection approach (see below). For each observed clinical patient encounter, the research assistants documented whether a given clinical process was performed by the health staff involved in the case according to national clinical guidelines, which were considered ‘gold standard’ for the purpose of this study. To cover large parts of the MNH Junction, observations started with the initial arrival of a laboring woman to the maternity unit and lasted until two hours after the delivery of the placenta (defined as immediate postpartum period for the purpose of this study). In order to include a sufficient number of delivery cases in each site, research assistants spent a minimum of three days at each individual facility and observed every delivery that took place during this period. Only cases in which both mid-level birth attendants and patients gave written consent to be observed were included into the study. The collected paper-based data were entered manually into an electronic dataset and analyzed using Stata software, version 12. Descriptive statistics were used to summarize the data. Frequencies and performance indices were used to summarize the observed clinical performance patterns. Performance indices were computed by assigning a score of 1 to each procedure observed as performed according to standard; the score for a non-performed procedure was 0. For each observed case a total performance score was calculated by summing the scores of each performed procedure relevant to s specific index. Mean performance scores were then computed by summing the total performance scores and dividing them by the number of observed cases. To allow easier comparability of the resulting performance indices, we divided each mean performance score by the number of procedures included in the index and transferred them into a relative 5-point index scale ranging from 0 (no performance) to 5 (complete performance). We created two types of performance indices: a risk-based index for each of the six identified direct mortality causes and a function-based index for each of the three routine care main functions. Ethical clearance for this study was obtained from the Ethical Committee of the Medical Faculty at the University of Heidelberg and from the College of Medicine Research and Ethics Committee.

### Selection of routine care indicators

**Fig 2:**
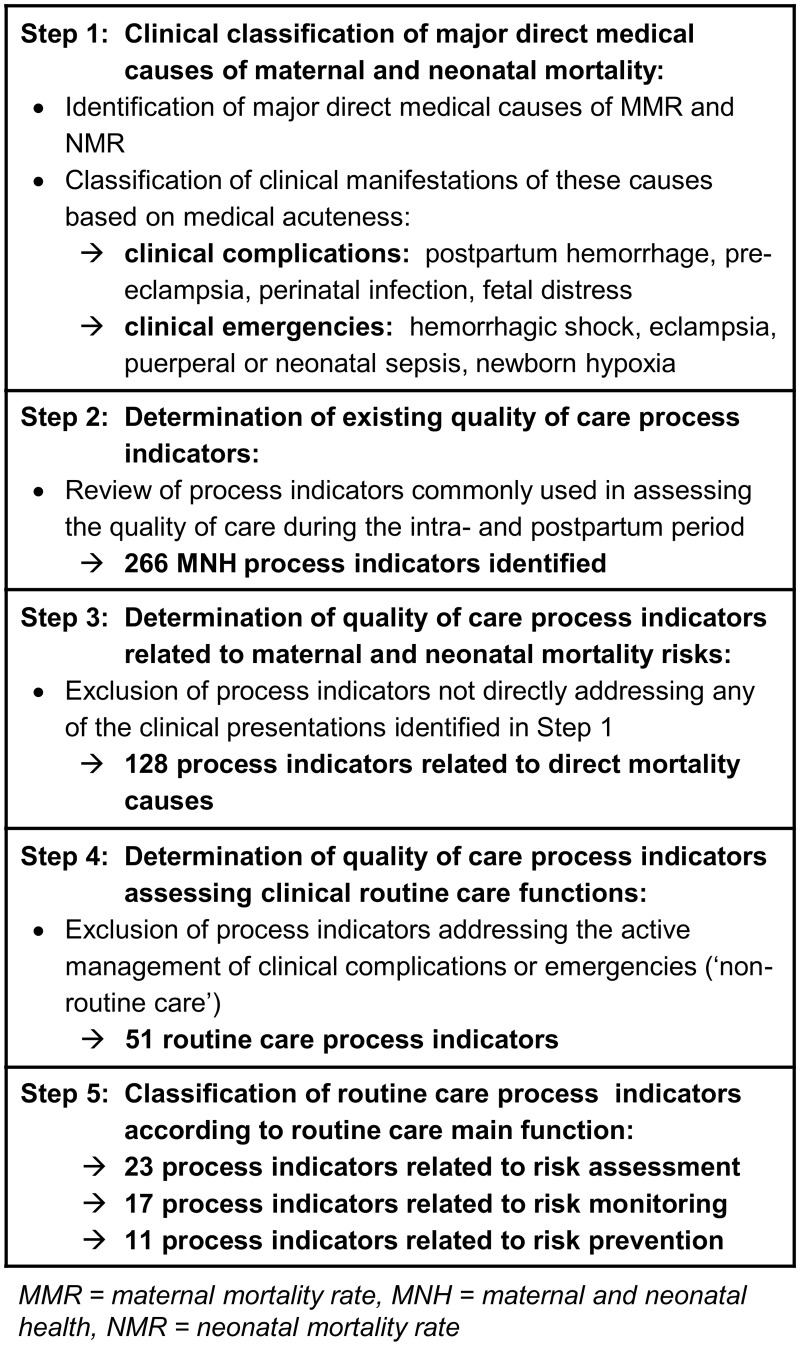
Overview of the steps leading to the selection of routine MNH care process indicators.

We only focused on quality of care indicators assessing clinical care processes [45][46]. The selection process consisted of five steps and is summarized in Fig 2.

In a first step, we identified the clinical presentations of major direct causes of maternal and newborn mortality (i.e. major obstetric and neonatal complications and emergencies) based on available epidemiological data [3,4,6,7,47]. We further classified possible clinical manifestations of the identified mortality causes based on their medical acuteness. For this purpose, we defined *medical complications* as any combination of abnormal clinical findings that constitute or expose a patient to a potential health risk. *Medical emergencies* were defined as the final clinical pathway of any unaddressed clinical complication leading to an acute life-threatening situation. We then identified those complications and emergencies most relevant to the MNH Junction, which included the following complications: PPH, pregnancy-induced hypertension or pre-eclampsia, infection of mother or newborn, forms of fetal or newborn distress; and the following emergencies: hemorrhagic shock, eclampsia, puerperal/neonatal sepsis, and birth asphyxia.

In a second step, we reviewed quality of care process indicators of different clinical MNH assessment tools and protocols developed for intrapartum- and immediate postpartum quality of care evaluation in LMICs [10,27–29,48] and further adjusted them, to the extent possible, to the Malawian context based on current national reproductive health quality assurance protocols and clinical training guidelines [49–53]. This allowed us to identify a total of 266 clinical process indicators relevant or commonly used in assessing MNH care quality.

In a third step, we reduced the list of clinical process indicators identified in step two by excluding indicators that addressed clinical aspects other than those identified in step one. The majority of indicators removed in this third step pertained to the prevention of mother-to-child transmission (PMTCT) of HIV, to aspects of patient centeredness, malaria prevention in pregnancy, and premature birth. The remaining 128 clinical process indicators are those directly addressing the epidemiologic profile of major maternal and neonatal causes of death (i.e. medical complications and emergencies) relevant to the MNH Junction.

In a fourth step, we used the definition of routine clinical care outlined above to further reduce the indicator list to contain only measures of obstetric and neonatal routine care processes. The majority of indicators removed in this fourth step pertained to the medical and surgical management of emergency conditions, such as treatment of eclamptic seizures, removal of retained products in hemorrhaging women, etc., as these processes would only be observed in a subset of women giving birth, and are also already covered in the EmOC signal functions. The resulting 51 indicators only measure clinical processes observable during every single provider-patient encounter at the MNH Junction regardless of the actual clinical case presentation.

In a last step, we assigned each of the 51 remaining clinical process indicators to one of three categories, each representing a main function of clinical routine care: 1) initial risk assessment; 2) continuous risk monitoring; and 3) potential risk prevention. These three functions are further explained below.

- The *initial risk assessment function* is essential to the beginning of a provider-patient encounter to allow a clinician an initial evaluation of the patient’s overall risk profile to further determine additional medical needs likely to be required in the later course. Generally, this function comprises indicators related to a patient’s medical history (i.e. assessment of clinical symptoms), as well as a focused physical examination (i.e. assessment of clinical signs). At the MNH Junction, this function includes the clinical assessment of a woman’s cardiovascular status (e.g. blood pressure, heart rate, anemia), her risk of infection or pregnancy-induced hypertension (e.g. time of membrane rupture, body temperature, chest auscultation, peripheral edema, urine protein), but also an initial evaluation of signs of fetal or neonatal distress (e.g. fetal heart rate check and intrauterine presentation, responsiveness and breathing effort of the newborn).
- As a clinical case continues, a patient’s risk profile requires repeated re-assessments—either in response to initially identified risk factors or in adherence to clinical protocols. Routine care processes related to the clinical re-evaluation of risk factors are include in the risk monitoring function. At the MNH Junction, this includes the monitoring of labor progression during the first labor stage (e.g. use of partographs to observe the dilation of the birth canal, the descent of the fetus, or the development of prolonged or obstructed labor), the continued monitoring of a woman’s cardiovascular status and infection risk (e.g. documenting maternal vital signs and temperature using a partograph, checking the appearance of amniotic fluid, immediate postpartum monitoring of maternal and neonatal vital and physical signs to detect PPH, immediate postpartum assessment of the effect of AMTSL), and the continued monitoring of the newborn during the immediate postnatal period (e.g. checking a newborn’s responsiveness and temperature).
- Elements of the *risk prevention function* occur throughout the provider-patient encounter and involve processes that actively reduce or control a patient’s risk of developing medical complications. At the MNH Junction, this includes processes of infection prevention (e.g. hand hygiene, use of sterile supplies and equipment, performance of certain examinations and other clinical procedures in a sterile manner) and PPH prevention (e.g. use of oxytocin and active delivery of the placenta during labor stage three as part of AMTSL), as well as the prevention of risk factors related to birth asphyxia and neonatal hypothermia (e.g. controlling fetal distress, removal of nuchal cord, suctioning airways, avoiding exposure of the newborn to a cool environment).

## Results

### Routine care process indicator selection

Our five-step selection rationale resulted in a set of 51 routine care processes (see Table 1). Divided into the three routine care main functions of clinical routine care, we identified 23 indicators related to the initial assessment of patient risk, 17 to risk monitoring, and 11 to risk prevention. In relation to the direct causes of mortality, 15 routine care indicators targeted a woman’s bleeding risk, and 11 addressed the mothers’ and newborns’ risk of perinatal infections. The risk of complications due to obstruction, and fetal distress were addressed by each 5 routine care indicators, whereas 7 indicators were related to the risk of newborn distress, and 8 indicators to the risk of pre-eclampsia. Maternal blood pressure checks were identified as a routine care process common to the prevention and monitoring of both the risk of pregnancy-induced hypertension/pre-eclampsia and bleeding. No routine care indicators specific to MNH Junction were identified for the prevention of prolonged labor based on our selection process.

**Table 1:** Final set of routine care process indicators by clinical core functions and major obstetric and neonatal complications.

	Initial Risk Assessment Function	Risk Monitoring Function*	Risk Prevention Function
**Postpartum hemorrhage**	Assessment anemia risk:	Monitoring bleeding risk during labor:	Controlling bleeding risk (AMTSL):
	• Verify history vaginal bleeding	• BP check hourly	• Prophylactic oxytocin administration
	• Check conjunctiva/palms	• HR check hourly	• Controlled cord traction
	• Check baseline hemoglobin level		• Uterus massage
			• Completeness check of placenta
	Assessment bleeding risk:	Monitoring bleeding risk immediately postpartum:	
	• BP check	• BP check hourly	
	• HR check	• HR check hourly	
		• Bleeding check hourly	
		• Uterine tone check hourly	
**Maternal and neonatal infection**	Assessment infection risk:	Monitoring infection risk:	Controlling infection risk (sterile practices):
	• Verify symptoms of infection	• Check maternal temperature 4-hourly	• Hand hygiene prior to direct patient contact
	• Verify time of ROM	• Check appearance of amniotic fluid 4-hourly	• Cleansing of perineum prior to vaginal exam
	• Temperature check		• Sterile equipment when performing invasive examinations
	• Chest auscultation		• Use of sterile equipment (blades, cord ties, gloves, etc.) during childbirth
	• Urine bacteria check		
**PIH and pre-eclampsia**	Assessment pre-eclampsia risk:	Monitoring pre-eclampsia risk during labor:	*(No relevant routine processes identified)*
	• Verify history of PIH	• BP check hourly	
	• Verify recent history of headaches		
	• Verify recent history of convulsions		
	• BP check		
		Monitoring pre-eclampsia risk immediately postpartum:	
		• BP check hourly	
		• Check for edema	
		• Urine protein check	
**Prolonged or obstructed labor**	Assessment labor progression:	Monitoring labor progression:	*(No relevant routine processes identified)*
	• Verify onset of labor	• Check contractions hourly	
	• Check fetal lie & position	• Check fetal decent check hourly	
		• Check cervical dilation 4-hourly	
**Fetal distress**	Assessment fetal condition:	Monitoring fetal condition:	Prevention fetal distress:
	• FHR check	• FHR check every 30 minutes during stage 1	• Check for & remove nuchal cord
	• Fetal movement check	• FHR check every 15 minutes during stage 2	
**Neonatal distress**	Assessment newborn’s condition:	Monitoring newborn’s condition:	Prevention neonatal distress:
	• Responsiveness check	• Temperature hourly	• Keep newborn dry and warm
	• Temperature check	• Responsiveness hourly	• Encourage skin-to-skin care
	• Weight check		

*) time intervals/frequency for specific monitoring processes as suggested by National Integrated Infection Prevention, Reproductive Health and PMTCT Performance Standards for Health Centres: Consolidation of the results of the Assessment Tool. (2010). Ministry of Health Malawi.

AMTSL = active management of third stage labor; BP = blood pressure; CV = cardiovascular; HR = heart rate; FHR = fetal heart rate; PIH = pregnancy-induced hypertension; ROM = rupture of membranes

### Testing of suggested process indicators

A total of 82 delivery cases were observed in 31 rural health facilities (45 observations in health centers, 37 observations in district hospitals). In two facilities no observations could be conducted due to the lack of deliveries during the research assistants’ stay. None of the 82 observed cases exhibited any obvious signs of clinical complications at initial presentation to the maternity ward. Given the different stages of labor at initial presentation and the variation in the sequence of clinical routine processes per each observed case, not all 51 identified processes could be observed in every single provider-patient encounter. This explains the differences in total numbers of observations for each process indicator. For the initial risk assessment function, the numbers of cases observed per indicator ranged from 66 to 78 (80–95% of all observed cases), for the risk monitoring function from 58 to 76 (71–93% of all observed cases), and for the risk prevention function from 61 to 76 (74–93% of all observed cases). The relatively low sub-sample of cases for the risk monitoring function is due to the fact that only 58 out of 82 cases (71%) were actively monitored by partograph during stage one labor, and only 76 out of 82 cases (93%) were kept in the maternity unit for direct immediate postpartum observation during the first two hours after delivery.

The results on single process indicator performance as well as details on indicator-specific sample sizes are summarized in Table 2. The scores of the performance indices for the examined risk factors and routine care functions are provided in Table 3.

**Table 2:** Observed frequencies of routine care processes by clinical core function and complication risk.

	Initial Risk Assessment Function	%	*N*	Risk Monitoring Function	%	*N*	Risk Prevention Function	%	*N*
**Bleeding Risk**	Anemia history	62	*73*	Hourly BP labor**	33	*58*	Oxytocin (AMTSL)	97	*76*
	Anemia exam	58	*66*	Hourly HR labor	31	*58*	Cord traction	82	*76*
	Hemoglobin lab	3	*73*	Hourly BP pp**	14	*76*	Uterine massage	96	*76*
	CV status BP*	56	*66*	Hourly HR pp	16	*76*	Placenta complete	93	*76*
	CV status HR	62	*66*	Hourly bleeding pp	11	*76*			
				Hourly uterus tone pp	12	*76*			
**Infection Risk**	Fever history	16	*78*	4-Hourly temperature	33	*58*	Hand hygiene	31	*61*
	Time of ROM	70	*69*	4-Hourly amniotic			Perineal cleansing	42	*61*
	Temperature check	52	*66*	fluid	31	*58*	Sterile exam	72	*61*
	Chest auscultation	12	*66*				Sterile delivery	85	*75*
	Urine bacteria lab	0	*73*						
**PIH/Pre-eclampsia Risk**	Headache	12	*73*	Hourly BP labor**	33	*58*	*(No relevant routine processes identified)*		
	Convulsions	12	*73*	Hourly BP pp**	14	*76*			
	Hypertension	26	*73*						
	CV status BP	56	*66*						
	Edema check	38	*66*						
	Urine protein lab	1	*66*						
**Obstruction Risk**	Onset contractions	84	*69*	Hourly contraction	36	*58*	*(No relevant routine processes identified)*		
	Fetal position	85	*66*	Hourly descent	31	*58*			
				4-Hourly dilation	32	*58*			
**Fetal Risk**	Fetal movement	64	*69*	30-min FHR labor	41	*58*	Nuchal cord check	84	*75*
	FHR check	94	*66*	15-min FHR birth	32	*75*			
**Neonatal Risk**	NB response check	91	*75*	Hourly response	8	*76*	Drying & warming	96	*75*
	NB temperature	8	*75*	Hourly temperature	3	*76*	Skin-to-skin care	92	*75*
	NB weight	96	*75*						

* identical process indicators of risk assessment for both bleeding and pre-eclampsia

**identical process indicators of risk monitoring for both bleeding and pre-eclampsia

AMTSL = active management of third stage labor; BP = blood pressure; CV = cardiovascular; FHR = fetal heart rate; HR = heart rate; min = minutes; N = total number of cases observed; pp = postpartum. % = frequency in percent of observed cases; PIH = pregnancy-induced hypertension; ROM = rupture of membranes

**Table 3:** Overall performance indices (using relative scale ranging from 0 = no performance to 5 = complete performance).

**Performance indices for risk factors based on direct causes of mortality:**	
Bleeding Risk	3.4
Infection Risk	2.5
Pre-eclampsia Risk	1.8
Obstruction Risk	3.2
Fetal Risk	3.4
Neonatal Risk	2.9
**Performance indices based on routine care main functions:**	
Risk Assessment	2.4
Risk Monitoring	2.2
Risk Prevention	4.2

Examining the performance quality of each of the three main functions of routine care (i.e. initial risk assessment, risk monitoring, risk prevention), our data showed the following:

#### Initial Risk Assessment Function

Overall, risk assessments were performed relatively incompletely as shown by a performance index of about 2 out of 5 (Table 3). Routine care processes related to the initial assessment of risks of prolonged labor and fetal or neonatal distress were more frequently performed according to clinical standards than the initial risk assessments of bleeding, infection and pre-eclampsia (Table 2). Of the processes related to the risk assessment of bleeding complications, hemoglobin checks were the least routinely performed in only 3% of observed cases. In comparison, processes pertinent in assessing the risk of pre-eclampsia and intrapartum infections varied widely and ranged from less than 1% of cases for urine-based diagnostics up to 70% for the determination of the time of membrane rupture.

#### Risk Monitoring Function

Similar to the previous, the risk monitoring function was also performed relatively incompletely with a performance index of about 2 out of 5 (Table 3). As shown in Table 2, risk monitoring for maternal or neonatal infections, prolonged labor, and fetal distress, as well as cardiovascular monitoring of the mother during labor were routinely performed in about a third of cases. Risk monitoring of immediate postpartum bleeding or hypertension (i.e. blood pressure measurement) and of neonatal distress represented the most infrequently observed processes in only 3 to 16% of cases.

#### Risk Prevention Function

The overall performance of preventive measures was relatively high as shown by a performance index of about 4 out of 5 (Table 3). Especially measures related to the prevention of immediate postpartum bleeding (i.e. AMTSL) and fetal and newborn distress (i.e. immediate newborn care) were performed according to clinical standards in about 90% or more of observed cases (Table 2). The least consistently performed risk prevention processes were related to routine infection control measures with observed frequencies for single indicators ranging from 31% for hand hygiene to 85% for sterile delivery conditions.

Examining the six major risk factors for maternal or neonatal complications and emergencies (i.e. bleeding, infection, pre-eclampsia, prolonged labor, fetal and neonatal distress) in respect to routine care performance, we found the following pattern:

#### Bleeding Risk

With a performance index of about 3 out of 5, the overall management of bleeding risks at the MNH Junction was of intermediate quality (Table 3). The observed frequency of routine care processes directly related to bleeding complications varied greatly (Table 2). Whereas initial risk assessment processes were consistently performed in about two thirds of cases (i.e. anemia and cardiovascular status of mother), consistent intrapartum monitoring of possible bleeding (i.e. cardiovascular status) was only observed in about one third of cases. In contrast, risk reduction of immediate postpartum bleeding (i.e. AMTSL) was provided in almost all cases, whereas the success rate these preventive measures during the immediate postpartum period (i.e. cardiovascular status, uterine contractions and actual bleeding) was only evaluated in 11–16% of cases.

#### Infection Risk

As shown in Table 3, routine care functions addressing maternal and fetal/neonatal infection risks were also performed with intermediate quality as demonstrated by a performance index of 2.5 out of 5. Altogether, routine detection and control of infection causes in mother or newborn were very inconsistently observed (Table 2). Besides inquiring on time of membrane rupture in 70%, no other possible infection sources with potential for intra- and postpartum complications were evaluated on a routine basis. Intrapartum monitoring of signs of infection (i.e. body temperature, color of amniotic fluid) was observed in only about 30% of cases. Although the consistent use of sterile equipment, such as sterile gloves and sterile delivery kits, was observed in 72% and 85% of cases respectively, the practice of hand hygiene and perineal cleansing was only performed according to clinical standards in 31% and 42% of cases respectively.

#### Pre-Eclampsia Risk

Routine care measures addressing risk factors of pre-eclampsia were of lowest performance quality with an index of about 2 out of 5 (Table 3). Routine evaluation of mothers’ risk of pre-eclampsia, such as the assessment of signs or symptoms of pregnancy-induced hypertension was only observed in a minority of cases (Table 2). Blood pressure checks to rule out pregnancy-induced hypertension were more common during the initial assessment (about half of observed cases).

#### Prolonged Labor Risk

Overall, performance quality related to potential labor obstruction risks was intermediate as demonstrated by a performance index of about 3 out of 5 (Table 3). While initial assessment of signs of prolonged labor or obstruction (i.e. onset of labor, fetal position and lie) was observed according to clinical standards in about 85% of cases (Table 2), risk monitoring during labor progression (i.e. partographing of contractions, fetal decent, cervical dilation) was only performed in about one third of observed cases.

#### Fetal Distress Risk

As shown by the intermediate performance index of about 3 out of 5 in Table 3. While assessment of fetal well-being (i.e. fetal movement, fetal heart rate) was observed in the majority of cases (64% and 94% respectively), monitoring for fetal distress during the intrapartum period (i.e. fetal heart rate checks during labor and delivery) was only observed in about one third of cases (41% and 32% respectively) (Table 2).

#### Newborn Distress Risk

The consistency with which routine care processes were used to address the risks of neonatal complications was also relatively variable (Table 2), which is reflected by the overall performance index of about 3 out of 5 in Table 3. In a majority of cases assessments of the newborn’s well-being (i.e. responsiveness, weight) and prevention of risk of neonatal distress (i.e. hypothermia prevention) were routinely observed according to clinical standards. However, routine monitoring of the newborn during the immediate postnatal period was only performed in very few cases.

## Discussion

### Identification of routine care process indicators

We identified a set of process indicators for the assessment of the quality of routine care in MNH by following a pre-defined selection strategy. In order to reduce the wide range of routine care processes currently available to an essential set of indicators, we relied on a selection process strongly oriented towards the direct causes of maternal and neonatal mortality. Aligning this focus on mortality causes with the strategic framework of the continuum of care, we identified the MNH Junction as the most critical point in time in the care of pregnant mothers and their newborns. Extracting MNH routine care processes within this defined scope resulted in a clearly framed set of key process indicators we consider essential to any MNH quality of care assessment.

Common methodologies used in the assessment of clinical care processes are medical record reviews, staff interviews, interviews with patients after having received care (i.e. exit interviews), equipment checks, and direct observation of care. Of these, direct observation is considered gold standard due to the relative high sensitivity and specificity this method offers in detecting poor clinical performance [54]. The identification of MNH ‘routine care signal functions’ feasible for direct observation was therefore our primary focus.

Some of the routine care indicators suggested here have also been shortlisted by other authors or are already included in tools used by initiatives like AMDD (Averting Maternal Death and Disability), DHS (Demographic and Health Surveys), or MCHIP [24,34–39], for instance correct partograph use and AMTSL. The difference between other approaches and ours is depth and/or breadth, as well as the underlying assessment strategy. The MCHIP surveys aim at providing a broad range of quality of MNH care measures with a high level of information detail. To do so comprehensively, not only direct observations but also inventory checklists, provider and patient interviews, and document reviews are necessary. The routine care signal functions suggested by Gabrysch et al. [24] are part of a comprehensive set of signal functions including not only routine care, but also emergency newborn care in addition to the established EmOC signal functions. The proposed six routine function are broadly framed clinical process indicators(e.g. monitoring and management of labour with partograph, using measures of infection prevention) that could be collected as part of in-depth facility assessments, but in particular lend themselves to inclusion in large-scale facility survey tools. The approaches to assess functionality can thus range from simple (e.g. reported provision) to sophisticated (direct observation).

In comparison, by taking direct observation as gold standard in our approach, we increased the depth of routine care measures by defining processes contained in broader care components in more detail (e.g. ‘hourly blood pressure checks’ as one process of the component ‘correct partograph use’). In addition, we reduced the assessment breadth by keeping focus on routine care processes relevant to the MNH Junction.

By outlining this approach and applying the resulting indicator set to an actual field setting we offer an example for a MNH quality of care assessment with focus on routine care processes relevant to the MNH Junction. We hope that this example encourages other MNH quality of care programs to include both the MNH Junction focus and the suggested routine care indicators more explicitly into future quality of care assessments. We expect that a wider application of our proposed approach will allow further refinement of the current indicator set and eventually achieve consensus on the use of routine care process indicators useful for MNH service evaluations in LMICs. Consistent assessments based on a core set of routine care processes across MNH programs will facilitate implementation and evaluation efforts at national and international levels. Commonly shared standards in assessing MNH routine care indicators across LMICs settings could thus contribute to successfully reducing maternal and neonatal mortality.

### Routine care performance quality

The findings yielded in the Malawian setting highlighted several gaps in the performance of obstetric and neonatal routine care at the MNH Junction. At first glance, our quality of care assessment demonstrated that none of the major mortality risks (i.e. bleeding, infection, pre-eclampsia, prolonged labor, fetal and neonatal distress) were approached by birth attendants in a sufficiently consistent way. Risk assessment, risk monitoring, and risk prevention functions did not appear to be systematically aligned, and clinical performance often deviated from the underlying clinical standards. This is mainly reflected by the relatively low or at best intermediate risk-based performance indices ranging between 1.8 and 3.4 points on a 5-point scale. With bleeding, infection, and hypertensive disorders together representing more than half of all direct causes of maternal deaths in SSA [3], and PPH and postpartum infection having been reported most common direct causes of maternal mortality in Malawi [55], this inconsistent routine care performance at the MNH Junction is worrisome.

With respect to the main functions of clinical routine care (initial risk assessment, risk monitoring, and risk prevention), our assessment showed that risk prevention processes were generally more routinely performed than initial risk assessment or risk monitoring processes. This is reflected by the function-based performance indices where risk prevention functions are almost completely performed (index of 4.2 on a 5-point scale). The negative consequences on maternal health outcomes due to health providers’ incomplete performance in the assessment and monitoring of risk factors has been previously reported in Malawi [55]. Improvements in the quality of risk assessment and monitoring functions of routine MNH care are therefore critical in the Malawian context and beyond.

As demonstrated by the set of process indicators identified with our approach, the risk assessment function as part of high quality routine MNH care relies heavily on laboratory testing (e.g. urine and blood tests), thorough evaluation of a patient’s past medical history (e.g. pregnancy-related danger signs), and the focused assessment and interpretation of physical signs (e.g. chest auscultation, maternal and fetal vital signs). Therefore, systematic performance of the risk assessment function on a routine basis is challenged whenever healthcare providers face shortages of supplies (e.g. laboratory test kits, stethoscopes, thermometers). Similarly, systematic performance of the routine MNH care monitoring function is heavily challenged by inadequate supply (e.g. partograph forms), but also by shortcomings in infrastructure (e.g. dedicated postpartum beds within the maternity ward) and staff (e.g. time constraints, lack of supervision of inexperienced staff). The chronic staff shortage and overcrowding of maternity units in Malawi [56], is likely to be a main contributor to the poor performance quality in routine risk assessment and monitoring at the MNH Junction, especially during labor and early postpartum periods.

Structural limitations aside, the MNH Junction provides healthcare providers with a unique opportunity for uninterrupted patient care. Based on the relatively low risk-based indices, especially for pre-eclampsia, perinatal infection, and neonatal distress, our findings also indicate that mid-level birth attendants might not fully embrace this opportunity to their and their patients’ advantage. For example, incomplete assessment of a patient’s pregnancy history regarding symptoms of pregnancy-induced hypertension or fever, the lack of basic practices of hand hygiene, or the inadequate use of partographs cannot be attributed to structural deficits alone. Previous assessments of MNH service delivery in Malawi already pointed at the relatively weak knowledge base in respect to certain aspects of routine obstetric care among qualified health workers in Malawi [57][58]. Improving medical knowledge—especially achieving a comprehensive understanding and interpretation of clinical interrelations—remains certainly a key element of mortality reduction at the MNH Junction. Major obstetric and neonatal complications and emergencies can only be detected and prevented if risk assessment, risk monitoring and risk prevention processes specific to a given complication are well aligned and integrated into a provider’s clinical decision-making. We understand each identified routine care process at the MNH Junction as essential in addressing a specific mortality risk. In the Malawian example, we observed some inconsistencies in certain clinical performance patterns. For example, although the PPH prevention through AMTSL was very frequently performed according to standard, follow-up monitoring of the effectiveness of this preventive intervention during the postpartum period was extremely infrequent and misaligned. Similarly, although birth attendants commonly used sterile equipment for infection prevention purposes, other preventive measures to control infection spread, such as hand washing, were rarely part of this routine. Based on other studies, routine use of oxytocin in third stage of labor has been popular with healthcare providers as it expedites third stage labor and thus shortens the time a midwife has to spend with a woman after delivery [56]. Inadequate hand-washing practices have been found to be due to providers’ personal inconvenience, lack of additional sanitary supplies, or insufficient hygiene protocols [59]. The extent to which such rather organizational factors can be applied to this Malawian sample will require further qualitative exploration.

Based on discussions with Malawian healthcare providers in preparation of the data collection instrument, non-abidance by some of the selected processes was expected beforehand in the Malawian context. For example, due to frequent supply chain limitations, laboratory testing of hemoglobin and urine protein levels are currently rarely performed on a routine basis, but rather on an ‘as indicated’ basis, in spite of the existing guidelines. Additionally, while routine checks for bacteriuria are suggested by some international guidelines, this process is currently not part of national standards in Malawi. Such discrepancies between recommended process indicators related to specific diagnostic laboratory and imaging procedures and common practice are found in many LMICs settings and contribute to shortcomings in quality of care. Similarly, optimal adherence to some of the identified routine care processes might be affected by causes beyond the clinician’s control, such as logistical or political constraints. For example, shortages in essential medicines and equipment, poor infrastructure, and inequitable geographic distribution of health resources has been an ongoing challenge to healthcare service delivery in Malawi [60][61]. Such context-specific limitations remain a common challenge to the comparability of performance standards across settings.

### Limitations

The set of process indicators suggested here is not intended to yield a comprehensive evaluation of a wide range of clinical indicators; neither is it sufficiently refined to provide a detailed assessment of MNH routine care aspects beyond the MNH Junction. What we attempt to achieve with the presented work is both to outline the need for a set of ‘signal functions’ for MNH routine care quality evaluations and to suggest one possible conceptual approach to address this need.

By prioritizing major causes of maternal and neonatal deaths, focusing on the MNH Junction within the continuum of care paradigm and aligning of processes along three main functions of clinical routine care, we attempted to identify process indicators that directly link non-emergency components of clinical care to mortality outcomes. With this focus we obviously excluded a number of other important routine care process indicators. We agree that, for instance, the role of patient-centeredness in modulating women’s health-seeking behavior [62], or the role of PMTCT in reducing the risk of intrapartum transmission of HIV in positive mothers [63] are essential to the quality of MNH care. We did not include these as we were not able to identify specific and strong direct links to the major mortality causes of women and their newborns in LMICs.

Although the identified process indicator set was sufficiently comprehensive to point out areas of low quality routine care performance at the MNH Junction, we faced limitations. For instance, when measuring the performance of monitoring functions, we rely on the providers’ documentation of process indicators, such as partograph entries. This is limited by the availability and use of clinical records in busy daily practice. Low scores on documentation-dependent indicators therefore need to be interpreted in the light of circumstances that might prevent healthcare providers from timely and complete record-keeping, and therefore might be underestimating actual clinical performance.

In respect to the Malawian example, the overall size of the observed sample in Malawi was relatively small. The purpose of this article, however, was to illustrate the application of our conceptual approach to routine MNH care, and therefore we considered this as secondary. Still, the Malawi findings outline a relatively clear pattern of shortfalls in the routine care provided at the MNH Junction, especially considering of the possibility of a Hawthorne effect and thus better performance than usual due to the observational assessment approach. For instance, the wide neglect of clinical monitoring or ineffective alignment of measures in PPH prevention provide some useful information to national and international health planners and policy makers in terms of clinical training and performance priorities. As long as there still persist relatively large deficits in MNH service delivery, concise but relevant sets of process indicators can provide sufficient information on the main shortcomings in addressing maternal and neonatal mortality.

## Conclusions

To inform the consensus on a set of ‘routine care signal functions’, we suggest an indicator selection rationale that aligns main functions of clinical routine care to direct causes of maternal and neonatal deaths. The set of process indicators identified by our approach is sufficiently precise to point at specific gaps in MNH service provision at the MNH Junction. Furthermore, in the Malawi example, the indicator set identified specific needs in clinical service delivery that should be addressed by policy reform: 1) mid-level birth attendants require further guidance in how to asses and monitor maternal risk factors; 2) mid-level birth attendants require further guidance in adequate monitoring of mother, fetus, and newborn throughout the MNH Junction; 3) mid-level birth attendants require further guidance in ensuring the effectiveness of preventive measures provided. To fully understand the underlying causes of these shortcomings, further quantitative and qualitative assessments on available inputs (i.e. human resource qualifications, supplies, service organization, provider motivation, etc.) is needed. We hope this approach to routine MNH care allows for further streamlining of midwifery protocols and quality assurance programs.
